# Promotion and Provision of Colorectal Cancer Screening: A Comparison of Colorectal Cancer Control Program Grantees and Nongrantees, 2011–2012

**DOI:** 10.5888/pcd11.140183

**Published:** 2014-10-02

**Authors:** Annette E. Maxwell, Peggy A. Hannon, Cam Escoffery, Thuy Vu, Marlana Kohn, Sally W. Vernon, Amy DeGroff

**Affiliations:** Author Affiliations: Peggy A. Hannon, Thuy Vu, Marlana Kohn, University of Washington, Seattle, Washington; Cam Escoffery, Emory University, Atlanta, Georgia; Sally W. Vernon, University of Texas Health Science Center at Houston, Houston, Texas; Amy DeGroff, Centers for Disease Control and Prevention, Atlanta, Georgia.

## Abstract

**Introduction:**

Since 2009, the Centers for Disease Control and Prevention (CDC) has awarded nearly $95 million to 29 states and tribes through the Colorectal Cancer Control Program (CRCCP) to fund 2 program components: 1) providing colorectal cancer (CRC) screening to uninsured and underinsured low-income adults and 2) promoting population-wide CRC screening through evidence-based interventions identified in the Guide to Community Preventive Services (Community Guide). CRCCP is a new model for disseminating and promoting use of evidence-based interventions. If the program proves successful, CDC may adopt the model for future cancer control programs. The objective of our study was to compare the colorectal cancer screening practices of recipients of CRCCP funding (grantees) with those of nonrecipients (nongrantees).

**Methods:**

We conducted parallel Web-based surveys in 2012 with CRCCP grantees (N = 29) and nongrantees (N = 24) to assess promotion and provision of CRC screening, including the use of evidence-based interventions.

**Results:**

CRCCP grantees were significantly more likely than nongrantees to use Community Guide-recommended evidence-based interventions (mean, 3.14 interventions vs 1.25 interventions, *P* < .001) and to use patient navigation services (eg, transportion or language translation services) (72% vs 17%, *P* < .001) for promoting CRC screening. Both groups were equally likely to use other strategies. CRCCP grantees were significantly more likely to provide CRC screening than were nongrantees (100% versus 50%, *P* < .001).

**Conclusion:**

Results suggest that CRCCP funding and support increases use of evidence-based interventions to promote CRC screening, indicating the program’s potential to increase population-wide CRC screening rates.

## Introduction

Colorectal cancer (CRC) is the second leading cause of cancer deaths among men and women in the United States (1). CRC screening reduces cancer deaths by detecting cancers at an early stage and by detecting and removing precancerous polyps before cancer develops (2). Screening is recommended for all adults aged 50 to 75 years (3). In 2012, 65.1% of adults in that age group met CRC screening guidelines, and 27.7% of the population had never been screened (4). People without health insurance or a regular health care provider were more likely never to have been screened (55% and 61%, respectively); however, a significant number with health insurance and with regular health care providers also remained unscreened (24% and 23.5%, respectively).

Studies suggest that most managers of cancer control programs are aware of the evidence supporting the effectiveness of CRC screening but are less familiar with research-tested (ie, evidence-based) interventions to increase CRC screening (5,6). In 2009, the Centers for Disease Control and Prevention (CDC) launched the Colorectal Cancer Control Program (CRCCP), which provides funding to 25 states and 4 tribes in the United States for 5 years. The CRCCP’s goal is to increase CRC screening rates among men and women aged 50 years or older in the funded states from its 2009 level of 64% to 80% by the end of 2014. CRCCP has 2 objectives. The first is to provide CRC screening to uninsured and underinsured men and women aged 50 to 64. CRCCP grantees use up to one-third of their funding to provide this screening. This objective is similar to that of CDC’s National Breast and Cervical Cancer Early Detection Program, which provides comprehensive cancer screening services to low-income, uninsured, and underinsured women. CCRP’s second objective is to promote population-wide CRC screening by using the evidence-based interventions identified in the Guide to Community Preventive Services (Community Guide, www.thecommunityguide.org). The interventions are both client-oriented and provider-oriented. Client-oriented interventions are small media (flyers, posters, and brochures), client reminders, and efforts to reduce structural barriers (eg, eliminating or simplifying administrative procedures; reducing wait time or distance between service delivery settings and target populations). Provider-oriented interventions are provider reminders and provider assessments, and feedback (7–11). CRCCP is the first CDC cancer screening program to encourage grantees to use evidence-based interventions to promote screening with the goal of having a population-level impact (12). CRCCP’s approach is consistent with the Institute of Medicine’s call for the engagement of multiple cross-sector partnerships between government public health and the broader community to promote and protect the public’s health (13).

CDC awarded CRCCP funds to 22 states and 4 tribal organizations in 2009 and funded 3 additional states in 2010. Over the first 4 years of the program, nearly $95 million was awarded to the grantee organizations. In 2013, award size ranged from $362,205 to $1,050,000. The CRCCP represents a new model for disseminating evidence-based interventions and promoting their use while also ensuring screening for a portion of the medically underserved. If successful, CDC may adopt the model for future cancer control programs. CDC is evaluating each aspect of the CRCCP, including grantees’ methods of providing screening and their use of evidence-based interventions to promote screening. The Cancer Prevention and Control Research Network (14) is one of CDC’s evaluation partners. Its mission is to accelerate the adoption of evidence-based cancer prevention and control strategies in communities. Studying whether and how the CRCCP model increases use of evidence-based interventions to promote CRC screening fits this mission. The Cancer Prevention and Control Research Network designs and fields an annual grantee survey that measures CRCCP grantees’ provision and promotion of screening.

Following the first grantee survey in 2011 (15), the evaluation team expanded its 2012 survey to include a comparison group of states and tribal organizations that did not receive CRCCP funding. This comparison group of states without CRCCP resources could use other funding sources for CRC screening and promotion, including the National Comprehensive Cancer Control Program and state-specific cancer initiatives. The objective of our study was to compare CRC screening provision and promotion activities in states and tribes that received CRCCP funds with the activities of states that did not receive funds. The purpose was to assess whether the CRCCP funding model was associated with grantee organizations’ greater use of evidence-based interventions for promoting screening than their use by organizations that did not receive CRCCP funding.

## Methods

Using DatStat Illume (DatStat Corp), we administered parallel online surveys to the 29 CRCCP grantees and to a comparison group of 33 state and tribal grantees that had National Breast and Cervical Cancer Early Detection Program funding but no CRCCP funding. Respondents were asked to report any CRC prevention and control activities. Both groups completed the surveys between September 28 and December 10, 2012. Survey questions were primarily closed-ended or Likert scale formatted, and they addressed CRC screening promotion and provision activities implemented from July 2011 through June 2012.

The survey included questions on use of 5 evidence-based interventions identified in the Community Guide (ie, small media, client reminders, reducing structural barriers, provider assessment and feedback, and provider reminders), reasons for using those interventions, and their ease of use. We also included questions to assess use of other strategies, including patient navigation (eg, transportation and language translation services), mass media, provider education, quality assurance and improvement activities, group and one-on-one education, and client or provider incentives. In regard to screening provision, the survey collected information on the type of screening test primarily used, recruitment strategies, and resources secured and partners enlisted for screening. The survey also included questions related to the use of patient navigation and to resources secured for cancer treatment. The University of Washington institutional review board declared the survey questionnaire and procedures exempt from review. Survey methods are more fully described by Hannon et al. (15). Descriptive analyses were performed from 2013 through 2014 using SPSS version 19 (IBM Corp). We report results from 2 sample *t* tests for continuous variables and χ^2^ tests and Fisher exact test for categorical variables by using *P* < .01 as a more stringent standard to limit the chances of spurious findings resulting from multiple comparisons. We also compared the practices of CRCCP grantee states and nongrantee states before CRCCP funding by using information from online sources and CDC reports to assess comparability of the 2 groups. Parallel information was not available from tribal organizations.

## Results

All CRCCP grantees (100%) and 24 of 33 nongrantees (73%) completed the survey. Most respondents in both groups represented state health departments (77%) or tribal organizations (19%) and served as program directors (51%) or program managers (43%), with no significant differences between groups. Sixty-two percent of all respondents had been involved in cancer control work for more than 5 years, and 75% of respondents of organizations not CRCCP-funded reported being currently involved in promoting and providing CRC screening.

All respondents in both groups had funding for the National Breast and Cervical Cancer Early Detection Program and National Comprehensive Cancer Control Program and thus had a long history (more than 20 years) in implementing breast and cervical cancer screening and in conducting extensive cancer control planning. CRCCP grantee states had significantly higher CRC screening rates before program initiation, and comparison states had higher levels of poverty and lack of insurance (Table 1 ). The 2 groups did not differ at baseline with respect to other variables such as population-based CRC screening programs or mandated insurance coverage for CRC screening. Most nongrantees had applied for CRCCP funding; however, actual or planned use of evidence-based interventions was neither a funding requirement nor an evaluation criterion that was considered in the application review and ranking.

**Table 1:** Comparison of States With or Without Grants for a Colorectal Cancer Control Program (CRCCP) Prior to Program Initiation (2009)

State Characteristics	States with CRCCP Grants (N = 25)	States Without CRCCP Grants (N = 18)	*P* Value^a^
	n (%)	n (%)	
**Area, sq m** (23)			
<42,774	6 (24.0)	8 (44.4)	.24
42,774 to 71,300	6 (24.0)	5 (27.8)	
>71,300	13 (52.0)	5 (27.8)	
**Population** (23)			
<2,790,136	7 (28.0)	7 (38.9)	.80
2,790,136 to 6,044,171	9 (36.0)	5 (27.8)	
>6,044,171	9 (36.0)	6 (33.3)	
**Percentage of population with income below federal poverty level** (24)			
<13.5%	8 (32.0)	6 (33.3)	.03
13.5% to 17.0%	10 (40.0)	4 (22.2)	
>17.0%	7 (28.0)	8 (44.4)	
**Percentage of population aged 18–64 y that is uninsured** (25)			
<17.5%	8 (47.1)	0 (0)	.03
17.5% to 22.9%	4 (23.5)	5 (45.5)	
>22.9%	5 (29.4)	6 (54.5)	
**Percentage of population aged 50–75 y up-to-date with CRC screening** (26)			
<61.8%	4 (16.0)	7 (38.9)	.01
61.8% to 67.4%	9 (36.0)	7 (38.9)	
>67.4%	12 (48.0)	4 (22.2)	
**Have CDC funding for CRC awareness and advocacy activities** (27)	9 (36.0)	6 (33.3)	.25
**Have population-based CRC screening programs^b^** (27)	11 (44.0)	10 (55.6)	.18
**Have mandated insurance coverage for CRC screening** (27)	13 (52.0)	13 (72.2)	.11
**Participate in Dialogue for Action stakeholder meetings or CRC Capacity Study** (27)	16 (64.0)	9 (50.0)	.16

Abbreviations: CRC, colorectal cancer; CDC, Centers for Disease Control and Prevention.

a *P* values are based on Fisher exact test.

b May not be statewide.

### Screening promotion

CRCCP grantees were significantly more likely than nongrantees to use Community Guide-recommended evidence-based interventions to promote CRC screening (average of 3.14 vs 1.25 interventions, *P* < .001) (Table 2). Most commonly used interventions were small media (eg, flyers, posters, and brochures); client reminders (eg, postcards, letters or greeting cards, telephone calls, text or e-mail messages); followed by efforts to reduce structural barriers (eg, eliminating or simplifying administrative procedures and other obstacles and reducing wait time or distance between screening locations and target populations); provider assessment and feedback at physician’s offices, provider groups, federally qualified health centers, and other clinics; and provider reminders in patient charts.

**Table 2:** Interventions to Promote Colorectal Cancer Screening Used by Colorectal Cancer Control Program (CRCCP) Grantees and Nongrantees (N = 24), July 2011–June 2012

Intervention	CRCCP Grantees^a^ (N = 29)	Non-Grantees^a^ (N – 24)	*P* Value^b^
**No. of Community Guide-recommended interventions^c^ used, mean (SD)**	3.14 (1.41)	1.25 (1.33)	<.001
Small media	28 (97)	12 (50)	<.001
Client reminders	22 (76)	5 (21)	<.001
Reducing administrative and other structural barriers	17 (59)	6 (25)	.01
Provider assessment and feedback	13 (45)	3 (13)	.008
Provider reminders	11 (38)	4 (17)	.08
**Other interventions in use or planned for next 12 months**			
Patient navigation for CRC screening promotion	21 (72)	4 (17)	<.001
Mass media	15 (52)	10 (42)	.48
Provider education, professional development, including physician-to-physician education	12 (41)	9 (37)	.78
Quality assurance, quality improvement, including academic detailing, performance monitoring	8 (28)	7 (29)	.90
Group education	7 (24)	6 (25)	.97
One-on-one education	5 (17)	5 (21)	.75
Client or provider incentives	4 (14)	4 (17)	.78
Other	5 (17)	3 (13)	.64

Abbreviations: CRC, colorectal cancer; SD, standard deviation.

a Values are n and % unless otherwise indicated.

b *P* values are based on χ^2^ test for categorical variables or 2-sample *t* test for continuous variables.

c www.thecommunityguide.org

Primary reasons cited by CRCCP grantee organizations for using Community Guide-recommended interventions to promote CRC screening were that they were evidence-based (28% of responses), were supported or promoted by CDC (12%), addressed an identified need (18%), and were easily implemented (12%). Primary reasons for using Community Guide-recommended interventions cited by nongrantees were that they were evidence-based (16% of responses), addressed an identified need (17%), and were supported by their organizations (15%). CRCCP grantees and nongrantees similarly rated the ease or difficulty of implementing Community Guide-recommended interventions. On a 5-point scale (1= very difficult, 2 = somewhat difficult, 3 = neutral, 4 = somewhat easy, 5 = very easy), patient-directed interventions were rated from 3.2 (reducing structural barriers) to 3.8 (small media), and provider-directed interventions were rated from 3.1 (provider assessment and feedback) to 3.5 (provider reminders) for all organizations combined.

Both CRCCP grantees and nongrantees implemented additional strategies not currently recognized by the Community Guide to promote CRC screening, including patient navigation, mass media, provider education, quality assurance and improvement activities, group and one-on-one education, and client or provider incentives. CRCCP grantees were significantly more likely to use patient navigation to promote CRC screening than were nongrantees, either by their own staff or by reimbursing providers for delivery of patient navigation services. Core navigation activities routinely offered as part of screening promotion by both CRCCP and nongrantees were educating patients about CRC screening methods and bowel preparation, assessing and addressing barriers to screening, scheduling CRC screening appointments, making reminder calls, assisting with transportation, providing language translation, providing child or elder care, identifying payment for screening, flagging charts for medical providers to promote screening, and following up with patients about results and next steps after screening. All other strategies were used to a similar extent by CRCCP and nongrantees.

### Screening provision

CRCCP grantees were significantly more likely than nongrantees to provide CRC screening (100% vs 50%, *P* < .001) (Table 3). Among CRCCP grantees, about half provided colonoscopy (n = 13) or sigmoidoscopy (n = 1) as a primary screening test, and half provided high-sensitivity guaiac fecal occult blood testing (n = 5) or fecal immunochemical testing (n = 10). Among nongrantees that provided screening (n = 12), 67% provided colonoscopy as the primary screening test and 33% provided fecal occult blood testing. None of the nongrantees provided sigmoidoscopy or fecal immunochemical testing as primary screening tests. Only 6 of the 24 unfunded sites (25%) reported receiving financial resources specifically dedicated to support CRC screening, ranging from $100,000 to $1,026,000 (median = $585,000). To deliver CRC screening, CRCCP grantees reported more partnerships with primary care clinics, endoscopy/gastrointestinal clinics, and federally qualified health centers than did nongrantees. Both were equally likely to use patient navigators to support screening provision.

**Table 3:** Interventions Used by Colorectal Cancer Control Program (CRCCP) Grantees (N = 29) and Nongrantees (N = 24) to Promote Colorectal Cancer Screening, July 2011–June 2012

Intervention	CRCCP Grantees, n (%)	Non-Grantees, n (%)	*P* Value^a^
**Provides any CRC screening test**	29 (100)	12 (50)	<.001
**Primary screening test provided**	**N=29**	**N=12**	—
Colonoscopy/sigmoidoscopy	13/1 (48)	8/0 (67)	.28
High-sensitivity fecal occult blood test or fecal immunochemical test	5/10 (52)	4/0 (33)	—
**Partners with another organization to provide screening, type of partner**			
Primary care clinics, excluding federally qualified health centers	20 (69)	5 (42)	.11
Endoscopy or gastrointestinal clinics	18 (62)	4 (33)	.10
Federally qualified health centers	17 (59)	4 (33)	.15
Other	10 (34)	5 (42)	.67
**Patient navigators support for screening**	23 (79)	8 (67)	.40
**Recruit patients for screening**	**N=29**	**N=24**	—
Brochures	25 (86)	13 (54)	.02
Flyers and information posted in clinics, hospitals, or health care centers	24 (83)	10 (42)	.002
Pre-reviewing patient records and charts to identify patients eligible for screening	14 (48)	5 (21)	.04
Tailored letters or communication from health care provider	10 (34)	3 (13)	.06
Other	10 (34)	4 (17)	.14
None	1 (3)	9 (38)	.003
**Are partners and resources currently supporting treatment for patients with diagnosed CRC?**			
Yes	26 (90)	12^b^(50)	.17
No	0 (0)	2 (8)	—
No patients with diagnosed CRC yet	3 (10)	10 (42)	—

Abbreviations: CRCCP, Colorectal Cancer Control Program; CRC, colorectal cancer; —, not calculated.

a *P* values based on χ^2 ^test.

b Of the 12 sites that supported treatment of diagnosed CRC, 11 provided CRC screening.

More CRCCP grantees than nongrantees engaged in patient recruitment for screening through brochures, letters or other communication from health care providers, flyers and posters, and review of patient records to identify patients eligible for screening. All CRCCP grantees and 11 of the 12 non-grantees providing CRC screening had secured treatment for anyone with a diagnosis of cancer via their screening services through partners or resources (eg, charity care at hospitals, cancer centers, individual doctors, programs for indigent patients).

## Discussion

### Screening promotion

The CRCCP is a new and innovative public health model that uses evidence-based interventions to promote CRC screening at the population level and, consequently, serves as a natural experiment to allow us to study the adoption of evidence-based interventions that are encouraged by a funder. Our results have implications beyond the CRCCP, because more and more funders are encouraging or requiring the use of evidence-based interventions (16). Our comparison of CRCCP grantees and nongrantees suggests that the CRCCP funding model may successfully promote the implementation of evidence-based interventions. CRCCP grantees quickly adopted this new model, which is different from the approach used in the National Breast and Cervical Cancer Early Detection Program, which is to provide screening only and not to promote screening. Compared with nongrantee organizations, CRCCP grantees were significantly more likely to implement evidence-based interventions that the CDC’s CRCCP recommended. Specifically, CRCCP grantees were twice as likely as nongrantees to use small media and 3 to 4 times more likely to use client reminders to increase community awareness of CRC screening guidelines and to increase community demand for CRC screening. The frequent use of small media and client reminders may be due to their ease of implementation and the fact that the CRCCP grantees are able to take the lead on the implementation of these activities (15). Fewer grantees used interventions directed at health care providers, such as provider assessment and feedback and provider reminders, which usually require collaboration with a partner organization. Although 2 to 3 times more CRCCP grantees than nongrantees used provider-oriented interventions, use could perhaps be increased by providing technical assistance on how to implement these interventions.

For the most part, both groups were equally likely to implement strategies not currently recognized by the Community Guide, such as professional development and quality assurance and improvement; these activities are components of the National Breast and Cervical Cancer Early Detection Program and contribute to high-quality screening and adherence to screening guidelines. Both CRCCP grantees and nongrantees frequently used patient navigators for both promoting and providing screening. Although patient navigation has not been reviewed as a unique intervention by the Community Guide and is therefore not one of the Community Guide-recommended evidence-based interventions, an increasing number of studies suggest that patient navigation is a useful way to promote CRC screening overall and among minority groups (17–19). Another strategy that was frequently used by both CRCCP and nongrantees was mass media such as television, radio, newspapers and billboards. Using funds for mass media promotion can be costly and is cause for concern. A recent review of studies examining the effect of mass media on breast, cervical, and colorectal cancer screening determined that there is insufficient evidence to support use of these media (11). More research is needed to evaluate the efficacy of mass media in promoting cancer screening, which may be difficult because mass media are often used in combination with other interventions to promote screening.

### Screening provision

Only 6 of the 12 nongrantees that reported providing screening reported that they had funding allocated specifically for CRC screening. The other 6 grantees may have used more general funds to provide CRC screening or were able to support screening in other ways. Not surprisingly, because they had dedicated CDC funding for CRC screening, CRCCP grantees were significantly more likely than nongrantees to provide that screening and were more engaged in recruiting patients for screening. About half of the grantees provided either fecal occult blood testing or fecal immunochemical testing as their primary screening test, and half provided colonoscopy.

For the time period surveyed, July 2011 through June 2012, CRCCP grantees provided a total of 12,669 CRC screening tests, including a combined 6,877 fecal occult blood tests and fecal immunochemical tests, 5,680 screening colonoscopies, and 112 sigmoidoscopies. Nationally, colonoscopy use has significantly increased over the past decade while screening via stool tests has declined (20,21). The National Colorectal Cancer Roundtable and others argue for increased use of stool tests given the higher cost and limited availability of colonoscopy (22). Recognizing that patients have preferences about test type, CDC recommends that health care providers identify and offer the tests that their patients will be most likely to complete (4).

### Limitations

We do not have information on nongrantees that did not respond to the survey, and it is possible that responders were more likely to be involved in CRC activities than nonresponders. If this is the case, our findings would underestimate the effect of CRCCP funding on screening promotion and provision of nongrantees compared with grantees. Also, respondents may not have been aware of all CRC activities in their states or tribes. Both CRCCP grantees and nongrantees have a range of resources for CRC screening, but grantees reported on only CDC-funded CRC screening promotion and provision activities. All information reported here is based on an administrator’s assessment of the program and was not verified by actual data. Because we have no data on use of evidence-based interventions before CRCCP funding, we cannot exclude the possibility that use of the interventions may have been higher among CRCCP grantees than among nongrantees at baseline. However, the large differences in use of these interventions between the 2 groups and the lack of differences in the use of other interventions support our conclusions.

### Conclusions

Given limited program resources, the total number of screening tests provided through the CRCCP is relatively small; however, given their low income and lack of adequate insurance, the people screened would probably have remained unscreened were it not for CRCCP. CRCCP grantees were more likely to implement evidence-based interventions promoting CRC screening than nongrantees, suggesting that CRCCP funding and support can increase use of evidence-based interventions. Both groups were equally likely to promote CRC screening in other ways. Our evaluators will continue to monitor CRC screening rates as well as CRC incidence and mortality in communities served by CRCCP grantees and compare the rates and incidence with those in communities served by nongrantees to assess the effect of evidence-based interventions at the population level.
